# Profile of sleep disturbances in patients with recurrent depressive disorder or bipolar affective disorder in a tertiary sleep disorders service

**DOI:** 10.1038/s41598-023-36083-7

**Published:** 2023-05-31

**Authors:** Panagis Drakatos, David O’Regan, Yingqi Liao, Constantinos Panayiotou, Sean Higgins, Renata Kabiljo, Joshua Benson, Norman Pool, Masoud Tahmasian, Andrea Romigi, Alexander Nesbitt, Paul R. A. Stokes, Veena Kumari, Allan H. Young, Ivana Rosenzweig

**Affiliations:** 1 Sleep Disorders Centre, Guy’s and St Thomas’ NHS Foundation Trust, London, UK; 2 Faculty of Life Sciences and Medicine, King’s College London, London, UK; 3 Department of Neuroimaging, Sleep and Brain Plasticity Centre, Institute of Psychiatry, Psychology and Neuroscience (IoPPN), King’s College London, De Crespigny Park, Box 089, London, SE5 8AF UK; 4 Department of Biostatistics and Health Informatics, Social, Genetic and Developmental Psychiatry Centre, Institute of Psychiatry, Psychology and Neuroscience, King’s College London, Denmark Hill, London, SE5 8AF UK; 5 Department of Neuropsychiatry, St George’s Hospital, South West London and St George’s Mental Health NHS Trust, London, UK; 6 Institute of Neuroscience and Medicine Research, Brain and Behaviour (INM-7), Jülich Research Center, Jülich, Germany & Institute for Systems Neuroscience, Medical Faculty, Heinrich-Heine University, Düsseldorf, Germany; 7 IRCCS Neuromed Istituto Neurologico Mediterraneo Pozzilli (IS), Pozzilli, Italy; 8 Department of Neurology, Guy’s and St Thomas’ NHS Foundation Trust, London, UK; 9 Department of Psychological Medicine, Centre for Affective Disorders, Institute of Psychiatry, Psychology & Neuroscience, King’s College London, London, UK; 10 Division of Psychology, Department of Life Sciences, & Centre for Cognitive Neuroscience, College of Health, Medicine and Life Sciences, Brunel University London, London, UK; 11 Department of Psychological Medicine, King’s College London & South London and Maudsley NHS Foundation Trust, Institute of Psychiatry, Psychology and Neuroscience, Bethlem Royal Hospital, Beckenham, UK

**Keywords:** Neuroscience, Physiology, Medical research, Neurology

## Abstract

Bidirectional relationship between sleep disturbances and affective disorders is increasingly recognised, but its underlying mechanisms are far from clear, and there is a scarcity of studies that report on sleep disturbances in recurrent depressive disorder (RDD) and bipolar affective disorder (BPAD). To address this, we conducted a retrospective study of polysomnographic and clinical records of patients presenting to a tertiary sleep disorders clinic with affective disorders. Sixty-three BPAD patients (32 female; mean age ± S.D.: 41.8 ± 12.4 years) and 126 age- and gender-matched RDD patients (62 female; 41.5 ± 12.8) were studied. Whilst no significant differences were observed in sleep macrostructure parameters between BPAD and RDD patients, major differences were observed in comorbid sleep and physical disorders, both of which were higher in BPAD patients. Two most prevalent sleep disorders, namely obstructive sleep apnoea (OSA) (BPAD 50.8.0% vs* RDD* 29.3%, *P* = 0.006) and insomnia (BPAD 34.9% vs RDD 15.0%, *P* = 0.005) were found to be strongly linked with BPAD. In summary, in our tertiary sleep clinic cohort, no overt differences in the sleep macrostructure between BPAD and RDD patients were demonstrated. However, OSA and insomnia, two most prevalent sleep disorders, were found significantly more prevalent in patients with BPAD, by comparison to RDD patients. Also, BPAD patients presented with significantly more severe OSA, and with higher overall physical co-morbidity. Thus, our findings suggest an unmet/hidden need for earlier diagnosis of those with BPAD.

## Introduction

Associations between sleep disturbances and affective disorders such as the Bipolar Affective Disorder (BPAD) and Recurrent Depressive Disorder (RDD) have been increasingly recognised^1–3^. Whilst sleep of inadequate quality often precedes a depressive episode and increases the risk for the development of affective disorders, mood of heightened negative or positive valence during wakefulness can also disrupt the normal sleep pattern^4^. Although disturbed sleep is considered to be a key symptom of psychiatric disorders in standard diagnostic manuals (i.e. Diagnostic and Statistical Manual of Mental Disorders-V (DMS-V)^5^ and International Classification of Diseases (ICD)^6^), the presentation of sleep disturbances remains heterogeneous in affective disorders. Furthermore, whilst recent studies suggest a disrupted homeostatic and circadian drive to sleep as the link between sleep disturbances and RDD^7^, the bidirectional relationship between sleep and BPAD has yet to be fully understood^8,9^. This is of note, especially given that sleep and circadian disturbances present the core symptoms across the lifespan of the patients, and likely serves a key role in the aetiology and the very course of BPAD^7,9–11^. Gaining further insight into the sleep phenotypes of BPAD and RDD may be useful for understanding the underlying pathophysiology of affective disorders, providing differential markers of these affective disorders, and hence influencing the choice of treatment modality to improve symptom severity in affective disorders.

To that end, an exploratory retrospective study of polysomnographic and clinical records of patients presenting to a tertiary sleep disorders clinic with affective disorders was conducted. We were not able to observe any significant differences in the sleep macrostructure parameters between BPAD and RDD patients, but major differences were observed in their presenting co-morbidity. Moreover, in our (real-world tertiary) patient cohort, BPAD was more strongly associated with two most prevalent sleep disorders, namely with obstructive sleep apnoea (OSA) and insomnia. Thus, we then set to further characterise any possible differences in the OSA phenotypes in patients with BAPD and RDD, especially those suggesting possible differing underlying neuromechanisms.

## Methods and materials

### Participant selection

A retrospective exploratory cross-sectional study of polysomnographic recordings of patients with clinically confirmed psychiatric diagnoses of BPAD and RDD^5,6^ by their referring clinical specialists, who were investigated between 2015 and 2019 at a large tertiary Sleep Disorders Centre (Guy’s Hospital (GSTT), London, United Kingdom), was conducted. The study was granted ethical approval by the GSTT Institutional Review Board on Human Research (Project No 9342, GSTT NHS); according to the strict national guidelines. The GSTT Institutional Review Board on Human Research waived the need of obtaining informed consent due to use of retrospectively ascertained anonymized data and due to the fulfilment of the following non-negotiable stipulations (1) the study protocol abided by the strictest patients’ data confidentiality and (2) it complied with all requirements of EU General Data Protection Regulation and with the Declaration of Helsinki regulations.

We identified sixty-three BPAD patients (32 female; mean age ± S.D.: 41.8 ± 12.4) and 126 age- and gender-matched RDD patients (62 female; 41.5 ± 12.8) (Table 1). All patients with a diagnosis or a previous history of BPAD or RDD confirmed in their medical records and based on the International Classification of Mental and Behavioural Disorders^6^ were screened and selected for the exploratory (real-life) study. Patients were in a presumed full or partial remission, and no additional mood and affective symptoms severity measures were available. Given the main aims of our study were to identify differences in the sleep phenotypes between patients with BPAD and RDD, firstly we identified all patients with BPAD that fulfilled inclusion and exclusion criteria. Subsequently, all patients with RDD that also fulfilled exclusion and inclusion criteria were sought, and matched 2:1 for age, gender, and date of study, to all identified BPAD patients. Eligibility of patients was further reviewed individually based on their medical records. Patients with a documented history of acute neurological or other psychiatric conditions were excluded from the study, e.g. those in the acute psychotic episode. Additional exclusion criteria were: aged ≤ 18 years old; a split-night polysomnography study (PSG: i.e. a baseline sleep study followed by continuous positive airway pressure (CPAP) titration performed in the second half of the night); central events consisting more than 50% of the AHI, past or current history of substance abuse; past history of head injury or trauma, neurodegenerative disorders (e.g. Parkinson’s disease).

**Table 1:** Demographic and polysomnographic characteristics of bipolar affective disorder (BPAD, n = 63) and recurrent depressive disorder (RDD, n = 126) patients. Group comparisons were conducted using one-way ANOVAs for parametric variables, Mann–Whitney U tests for non-parametric variables and Pearson’s χ^2^ tests for categorical variables.

	BPAD		RDD		Statistics	
	Mean	SD	Mean	SD	F	*P* value
Age (years)	41.8	12.4	41.5	12.8	0.0180	0.893
BMI (kg/m^2^)	32.9	7.98	28.6	6.34	**15.379**	< 0.001*
	n	%	n	%	χ^2^	*P* value
Gender (female)	32	50.8	62	49.2	0.0420	0.837
	Median	IQR	Median	IQR	U	*P* value
ESS	12.0	13.0	14.0	9.0	3407.0	0.888
Sleep macrostructure						
TST (min)	379	119	369	117	3622.5	0.911
WASO (min)	53.7	86.0	74.4	74.0	4093.0	0.195
SL (min)	17.0	30.5	15.8	26.8	3725.5	0.845
REM L (min)	85.5	130	119	121	3761.0	0.203
NREM1%	9.24	11.0	11.4	8.73	3915.0	0.445
NREM2%	41.3	13.8	42.3	11.1	3916.0	0.607
NREM3%	27.3	17.9	25.2	12.8	3226.5	0.194
REM%	16.6	11.2	18.9	10.5	4265.5	0.070
AI	17.8	18.6	20.3	16.0	4082.5	0.096
AHI	5.40	11.0	1.70	5.90	**2802.5**	0.010*
PLMI	9.25	24.6	14.2	25.1	3806.5	0.105

Significant values are in bold.

%, percentage; AH, apnea–hypopnea; AHI, apnea–hypopnea index; AI, arousal index; BMI, body mass index kg/m^2^; D, relative density; ESS, Epworth Sleepiness Scale; F, one-way ANOVA F statistics; IQR, interquartile ranges; n, number; NREM , non-rapid eye movement; min, minute; REM, rapid eye movement; time; REM L, rapid eye movement latency; SL, sleep latency; SD, standard deviation; TST, total sleep time; WASO, wake after sleep onset; U, Mann–Whitney U statistics.

*P < 0.05.

### Polysomnography acquisition

All subjects had undergone overnight PSG, and the subsequent sleep scoring were performed in accordance with American Academy of Sleep Medicine (AASM) by two experienced sleep technologists^12^. The PSG comprised multiple channels: electroencephalogram (EEG, six channels), electrooculogram (EOG, two channels), chin, tibialis anterior electromyogram (EMG), electrocardiogram (ECG), pulse oximeter, thoracic and abdominal respiratory bands, position sensor, nasal flow cannula and infrared video recording^13^.

Sleep parameters included sleep architecture related variables (i.e. non rapid eye movement sleep stage 1 (NREM1), or rapid eyer movement sleep stage (REM) as a percentage of total sleep time (TST); NREM1% of TST, REM% of TST), sleep continuity variables (i.e. TST, sleep onset latency, i.e. the amount of time between the first epoch of any sleep stage and the ‘lights out’); REM onset latency, REML (i.e. the time taken to the first epoch of REM sleep after sleep onset); sleep efficiency, SE (i.e. total sleep time/total time in bed × 100); wake after sleep onset (WASO); AI (i.e. number of arousals/TST); periodic limb movements index, PLMI (i.e. number of periodic limb movements/TST).

The sleep apnea metrics included the apnea hypopnoea index (AHI, number of apneas + number of hypopneas)/TST), the supine- and non-supine-AHI, and the AHI related to the main sleep stages (AHI_NREM_, AHI_REM_). We also measured the proportion of the AHI of obstructive events that consists of obstructive hypopneas (HI/AHI) for the total sleep time, and for the NREM and REM sleep separately. A formula of Relative Density, AHI_(sleep stage)_/AHI_(total)_, or AHI_(sleeping position)_/AHI_(total)_ was also created to describe the subtypes of sleep apnoea by taking account of the sleep stage, and the sleeping position and its relative duration in total sleep time. A value > 1 indicates the predominance of sleep apnoea type during the night. For assessing sleep stage’s and body position’s contribution to the total number of apneas and hypopneas, we generated their respective ratios, i.e. number of apneas and hypopneas from NREM/total number of apneas + hypopneas (AH_NREM_/AH).

### Sleep disorders, other comorbidity, pharmacotherapy

Information regarding sleep disorders, other comorbid conditions and medications was retrieved from medical records. Diagnosis of sleep disorders was made by consultant sleep physicians, based on International Classification of Sleep Disorders third edition (ICSD-3)^14^. Recorded sleep disorders were subsequently included in further analysis. For statistical analysis, sleepwalking, sleep talking, sleep terrors, slow wave arousal disorders and other non-REM parasomnias were categorised as NREM parasomnia^15^, while supine- or REM-related OSA was included under the umbrella diagnosis of OSA. Moreover, unspecified sleep disorders (i.e. no identified organic sleep disorder from PSG but with a noted difficulty falling asleep or maintaining sleep), mild insomnia-like presentations with behaviourally induced insufficient sleep syndrome were both labelled as ‘poor sleep quality’ (PSQ) in the data analysis. Daytime sleepiness and excessive daytime somnolence was captured by the self-administered questionnaire Epworth Sleepiness Scale (ESS)^16^.

Medical records were searched for the diagnosis and medical history of other physical and psychiatric disorders and pharmacotherapeutic treatment plans. Subtypes of several disorders were grouped into one category for further analysis (e.g. personality disorder, chronic pain and arthritis). Another example is if a Nissan fundoplication was indicated in patients’ medical records, a medical history of gastro-oesophageal reflux disease would be noted as one of patient’s diagnoses. Additionally, individuals with body mass index (BMI) ≥ 30 were considered as obese^15^, which was then listed as comorbidity in our study.

### Statistical analyses

Data was recorded and subsequently analysed using the statistical software IBM SPSS Statistics Version 25 (IBM Corp., 2017). Means (standard deviations) or medians (interquartile ranges, IQRs) were reported as descriptive statistics for continuous variables, while numbers of samples (percentages) were reported for categorical variables. When the assumption of normality was met, independent sample t-tests were then used to compare continuous variables between the BPAD and RDD group, and χ^2^ tests for the comparison of categorical variables. For non-parametric variables, Mann–Whitney U tests and Fisher’s exact tests were conducted as appropriate. If differences were observed in variables between both groups, subgroup analyses were carried out for further investigation. Regression analysis with backword elimination and natural log transformation of the dependent variable HI/AHI was performed, with independent factors the AHI, AHI_NREM_, WASO, N1%, N2% and TST, following exploratory results from Pearson corelation between HI/AHI, BMI, the sleep parameters and OSA severity metrics. The statistical significance level was set at *P* < 0.05.

## Results

Socio-demographic and polysomnographic descriptive statistics is shown in Table 1. Sixty three BPAD patients, of whom 50.8% were women (N = 32), along with 126 RDD patients (49.2% women) who met the inclusion criteria, were investigated in the final analysis (see Table 1). The age of selected patients ranged from 18 to 66 years old, with a mean age of 41.8 (SD = 12.4) and 41.5 (SD = 12.8) years old in BPAD and RDD group, respectively (Table 1). BPAD patients’ BMI was significantly higher than that of RDD patients’ (32.9 ± 7.98 versus 28.6 ± 6.34, *P* < 0.001, Mann–Whitney U test) (Table 1). Conversely, there was no significant difference in daytime sleepiness between two groups (ESS score: BPAD: 12.0 ± 13.0 versus RDD: 14.0 ± 9.0, *P* = 0.888, one-way ANOVAs) and in majority of other sleep parameters (Table 1).

BPAD patients presented with overall higher sleep (Table 2) and non-sleep comorbidity (Supplementary Table 1) by comparison to RDD cohort.

**Table 2:** Comparison of obstructive** s**leep apnea metrics in obstructive sleep apnoea patients, and distribution of comorbid sleep disorders between patients with Bipolar Affective Disorder and patients with Recurrent Depressive Disorder. Group comparisons for categorical variables were conducted using Pearson’s χ^2^ tests for parametric variables and Fischer’s Exact tests for non-parametric variables, and for continuous variables, the Independent-Samples Mann–Whitney U test.

Sleep disorders	BPAD		RDD		χ^2^	*P* value
	n	%	n	%		
OSA	**32**	**50.8**	**37**	**29.3**	**19.450**	0.006**
Gender (female)	17	53.1	17	45.9	0.354	0.552
	Median	IQR	Median	IQR	U	
Age (years)	44.8	11.5	49.7	9.9	739.5	0.076
BMI (kg/m^2^)	**35.9**	**8.5**	**30.6**	**6.5**	**338.5**	0.004*
ESS	14.0	7	13.0	9.0	521.5	0.846
AHI (e/h)	10.0	12.7	10.7	7.1	515.5	0.357
HI/AHI (e/h)	0.81	0.25	0.78	0.27	538.5	0.520
AHI_REM_ (e/h)	**19.5**	**23.3**	**10.4**	**12.3**	**419.0**	0.037*
AHI_REM_/AHI (e/h)	1.67	2.03	1.44	1.61	357.0	0.086
AHs_REM_/AHs (n)	0.25	0.39	0.19	0.40	525.0	0.420
HI/AHI in REM (e/h)	0.83	0.52	0.79	0.48	548.0	0.596
AHI_NREM_ (e/h)	8.9	12.2	9.4	9.6	585.0	0.933
AHI_NREM_/AHI (e/h)	**0.85**	**0.63**	**0.94**	**0.24**	**764.5**	0.038*
AHs_NREM_/AHs (n)	**0.71**	**0.60**	**0.81**	**0.40**	**761.0**	**0.042**
HI/AHI in NREM (e/h)	0.84	0.24	0.82	0.36	535	0.492
AHI_Supine_ (e/h)	16.8	27.5	15.7	16.8	573.0	0.819
AHs_Supine_/AHs (n)	0.67	0.51	0.59	0.71	545.5	0.575
AHI_Non-supine_ (e/h)	5.1	5.8	5.4	9.2	570.0	0.791
AHs_Non-supine_/AHs (n)	0.32	0.51	0.41	0.71	645.0	0.523
Other sleep disorders	n	%	n	%	χ^2^	
Insomnia	**22**	**34.9**	**20**	**15.0**	**8.816**	0.005*
PLMD	9	14.3	23	18.3	0.470	0.544
NREM parasomnia	5	7.90	10	7.90	0.000	1.000
RLS	5	7.90	8	6.30	–	0.763
Idiopathic hypersomnia	4	6.30	11	8.70	0.326	0.777
PSQ	**3**	**4.80**	**32**	**25.4**	**12.507**	0.001*
Narcolepsy (I + II)	1	1.60	5	4.00	–	0.665
EFM	1	1.60	1	0.80	–	1.000
Sleep paralysis	1	1.60	0	0	–	0.333
DSPS	0		1	0.80	–	1.000
Drug-induced hypersomnolence	0		1	0.80	–	1.000
Catathrenia	0		1	0.80	–	1.000
Bruxism	0		1	0.80	–	1.000
CSA	0		1	0.80	–	1.000

Significant values are in bold.

%, percentage; AHs, total number of apneas and hypopneas; AHs_REM_, number of apneas and hypopneas during REM sleep; BPAD, bipolar affective disorder; CSA, central sleep apnea; DSPS, delayed sleep phase syndrome; EFM, excessive fragmentary myoclonus; e/h, events per hour; HI/AHI, hypopneas index to apnea and hypopneas index; n, number, NREM parasomnia, non-rapid eye movement parasomnia; NREM, non-rapid eye movement sleep; OSA, obstructive sleep apnea; PLMD, periodic limb movement disorder; PSQ, poor sleep quality; RDD, recurrent depressive disorder; REM, rapid eye movement sleep; RLS, restless legs syndrome.

*P < 0.05.

### Objective sleep measures

A total of 189 reports (Table 1) were analysed and variables of sleep architecture and sleep continuity were compared between the studied groups. No significant differences were noted in the sleep macrostructure. Patients with BPAD and RDD demonstrated a similar duration of sleep (TST: 379 ± 119 versus 369 ± 117 min, *P* = 0.911, Mann–Whitney U test). Moreover, our analysis showed that patients with BPAD and RDD experienced a similar level of arousals during the PSG (17.8 ± 18.6 versus 20.3 ± 16, *P* = 0.096, Mann–Whitney U test). However, a greater AHI was recorded in BPAD patients in comparison to RDD patients (5.40 ± 11.0 versus 1.70 ± 5.90, *P* = 0.01, Mann–Whitney U test), which reflected a greater prevalence of OSA in patients with BPAD relative to patients with RDD (Table 2**).**

### *Co-morbid sleep disorders*

**Figure 1:**
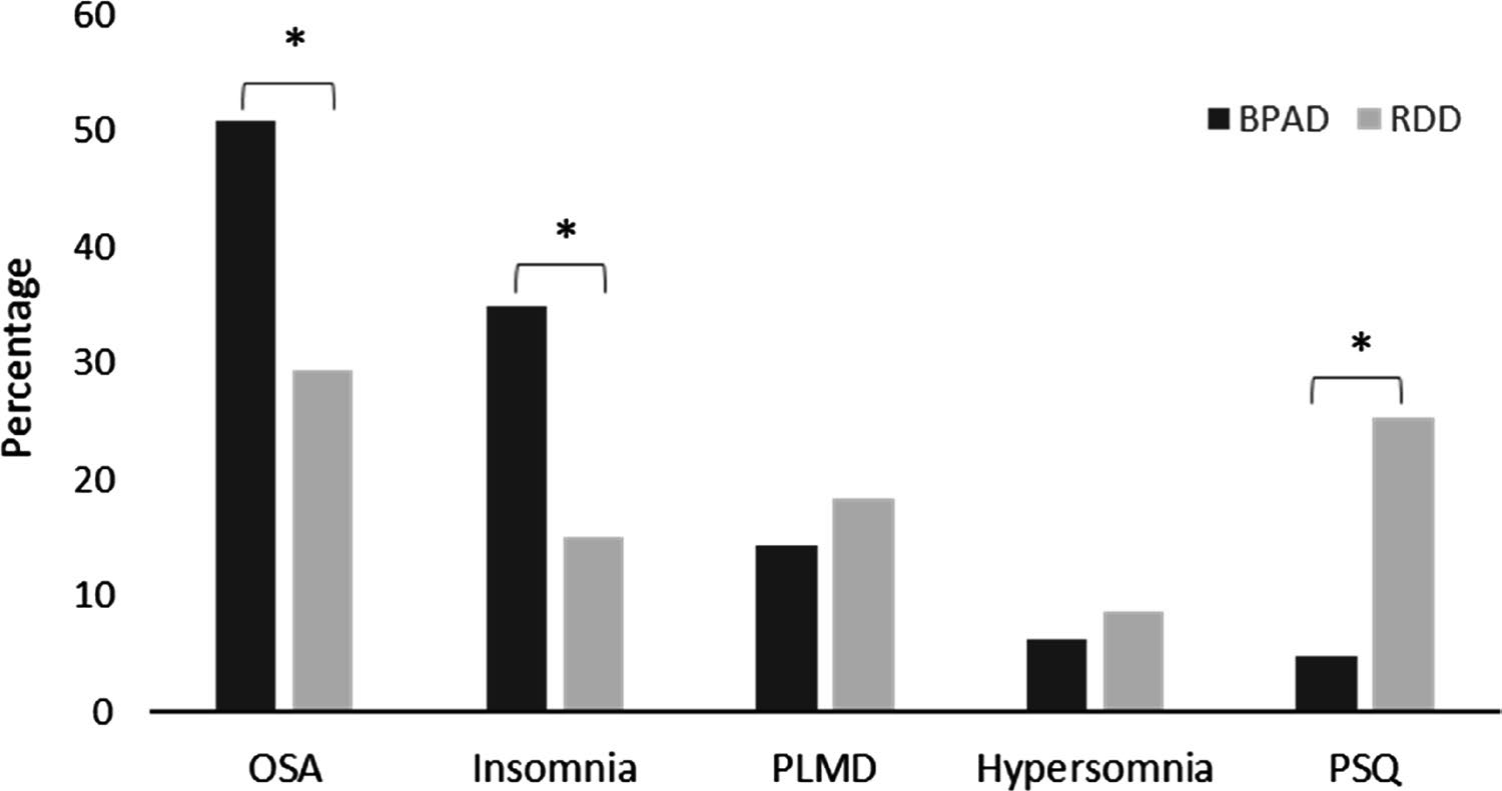
Most prevalent sleep disorders in bipolar affective disorder (BPAD) and recurrent depressive disorder (RDD). Analysis was performed using Pearson’s χ^2^ test. *PLMD* periodic limb movement disorder, *PSQ* poor sleep quality. *P < 0.05, χ^2^ test.

Only one patient (RDD) from our cohort presented with more than five events per hour of central sleep apnoea, and was thus excluded as per our eligibility criteria (CSA > 50% of the AHI). In both studied groups, OSA was the most prevalent comorbid sleep disorder among the seventeen sleep disorders recorded (Table 2). However, OSA was more frequently observed in BPAD patients relative to RDD patients (50.8% versus 29.3%, *P* = 0.006, Pearson’s χ^2^ test) (Table 2 and Fig. 1). Similarly, patients with BPAD were more often diagnosed with insomnia compared to patients with RDD (34.9% versus 15.0%, *P* = 0.005, Pearson’s χ^2^ test). Conversely, the analysis of medical records and PSGs of patients with RDD demonstrated that they more often suffered with a poor sleep quality (PSQ; Table 2), which upon objective analysis failed to reach the level of caseness for diagnosis of insomnia, or any other sleep disorders. The findings for other sleep disorders are also shown in Table 2.

**Figure 2:**
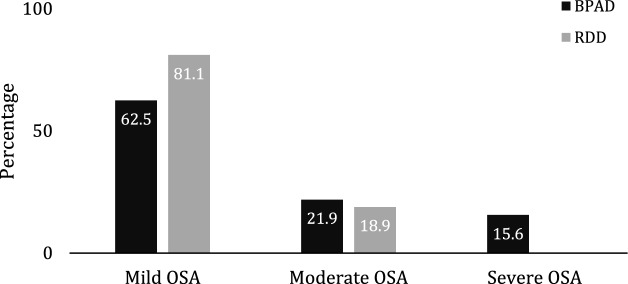
Graphical representation of the different distribution of obstructive sleep apnea (OSA) severity in patients with Bipolar Affective Disorder (BPAD) and Recurrent Depressive Disorder (RDD). The distribution of the OSA severity is statistically different between the two groups, *P* = 0.036 (Chi-Square test). Mild OSA, AHI 5–14.9 e/h; Moderate OSA, AHI 15–29.9 e/h; Severe OSA, > 30 e/h.

We then set to investigate OSA as the most prevalent sleep disorder in our cohorts. The further analyses did not reveal any significant differences in overall AHI of the OSA patients between the two groups (*P* = 0.357, Mann–Whitney U test). However, BPAD patients presented with higher OSA severity than RDD patients (Fig. 2, *P* = 0.036, Pearson’s χ^2^ test). Both cohorts presented predominantly with OSA of mild severity (BPAD 62.5% versus 81.1% RDD, Fig. 2). The BPAD group had higher AHI in REM sleep (*P* = 0.037, Mann–Whitney U test), however, the relative density (AHI_REM_/AHI) and the relative contribution to the total number of events (AHs_REM_/AHs) was comparable between the two cohorts (*P* = 0.086 and *P* = 0.420, Mann–Whitney U test, respectively; Table 2).

Of note, RDD patients presented with the higher relative density of OSA during NREM (AHI_NREM_/AHI), and the respective contribution to the overall events (AHs_NREM_/AHs), was recorded as significantly higher in the RDD group (*P* = 0.038 and *P* = 0.042, Mann–Whitney U test, respectively; Table 2), perhaps suggestive of higher mechanistic vulnerability of this cohort to respiratory events during NREM sleep.

**Figure 3:**
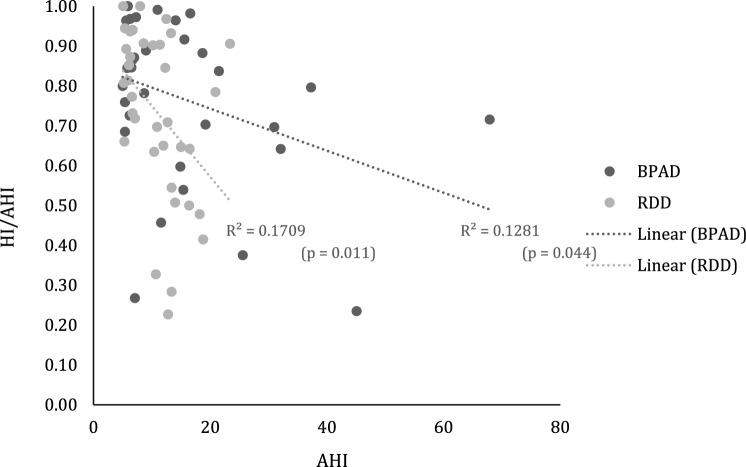
Scatter plot with best fit regression lines between the HI/AHI ratio and the AHI for patients with Bipolar Affective Disorder (BPAD) and Recurrent Depressive Disorder (RDD). *HI/AHI* hypopneas index to apnea and hypopnea index.

Of note is that in 75% of all patients, hypopneas dominated in almost 2/3 of all respiratory events. No differences were recorded between groups in the hypopnea index (HI) as fraction of the AHI (HI/AHI) in any of sleep stages (Table 2). In addition, correlational analyses suggested link between (a) overall milder OSA severity (BPAD: r = − 0.359, *P* = 0.044 and RDD: r = − 0.413, *P* = 0.011, Pearson correlation, respectively; Fig. 3), (b) milder OSA during NREM (BPAD: r = − 0.397, *P* = 0.025, and RDD: r = − 0.495,* P* = 0.002, Pearson correlation, respectively), and (c) the higher percentage of NREM2 sleep (BPAD: *r* = 0.474, *P* = 0.006, and RDD: *r* = 0.339, *P* = 0.040, Pearson correlation, respectively), and higher hypopnea prevalence in both cohorts (Supplementary Table 2). For RDD patients, an additional link was seen with reduced amount of time spent awake after falling asleep (WASO: r = − 387, *P* = 0.018, Pearson correlation) and with a duration of sleep (TST: r = 0.457, *P* = 0.004, Pearson correlation; Supplementary Table 2). No associations were found between hypopneas and daytime somnolence, or with demographic descriptive in either group.

For BPAD patients, a significant association was found between BMI and AHI (r = 0.555, *P* = 0.001), AHI_NREM_ (r = 0.456, *P* = 0.010), and AHI_REM_ (r = 0.361, *P* = 0.046), but not with HI/AHI (r = 0.043, *P* = 0.818), nor for any of the sleep stages (*P* > 0.05). There were no significant links with the AHI or HI indices in RDD group (Supplementary Table 2).

Regression analyses showed that for the BPAD patients, decreasing AHI_NREM_ (− 0.391, *P* = 0.012) and increasing NREM2% of TST (0.469, *P* = 0.003) were the sole two independent predictors of HI/AHI variance (r^2^ = 0.378, P = 0.001). In line with other analyses, for RDD patients, the model was statistically significant for predicting 41% of the HI/AHI variance (r^2^ = 0.409, *P* < 0.001), sharing with BPAD the AHI_NREM_ factor (r^2^ = − 0.407, *P* = 0.006) and the NREM2% of TST (r^2^ = 0.248, *P* = 0.078) factors, in addition to WASO (r^2^ = − 0.320, *P* = 0.024).

### Other (non-sleep) co-morbidities

Descriptive statistics for other (non-sleep) comorbid conditions are shown in the Supplementary Table 1. In total, 75 comorbid conditions were recorded in the current study. Obesity (BMI ≥ 30) was the most prevalent comorbid condition in both patient groups, with a significantly greater prevalence in individuals with BPAD compared to individuals with RDD (52.4% versus 31.0%, *P* = 0.007, Pearson’s χ^2^ test) (Supplementary Table 1). In the BPAD group, the second most frequent comorbid conditions were jointly diabetes (14.3% versus 3.2%, *P* = 0.011, Fischer’s Exact test) and hypothyroidism (14.3% versus 2.4%, *P* = 0.003, Fischer’s Exact test), while hypertension was frequently reported in both groups (7.9% versus 7.9%, *P* = 1.00, Fischer’s Exact test).

Furthermore, Fisher’s exact tests revealed that several disorders were significantly more common in patients with BPAD than in patients with RDD, including asthma (11.1% versus 3.2%, *P* = 0.044), irritable bowel syndrome (IBS) (7.9% versus 0, *P* = 0.004), fibromyalgia (6.3% versus 0.8%, *P* = 0.043), migraine (6.3% versus 0.8%, *P* = 0.043), attention deficit hyperactivity disorder (ADHD) (4.8% versus 0, *P* = 0.036) and hyperlipidaemia (4.8% versus 0, *P* = 0.036).

### Pharmacotherapy

Patients with BPAD and RDD differed in the number of medications taken (*P* = 0.006, Fischer’s Exact test). The majority of patients with BPAD were prescribed at least two medications (17.5%), whereas patients with RDD were mostly treated by only one medication (32.5%). Furthermore, the number of medications taken by patients significantly varied, from no medication (6.30% versus 13.5%) to a combination of more than 15 medications (one patient in each group). Most frequently recorded pharmacological treatments for two cohorts are shown in Supplementary Table 3.

The most commonly used medication in the BPAD group was lamotrigine, an anticonvulsant and a mood stabilising medication, which was prescribed significantly more frequently than in the RDD group (27.0% versus 0.80%, *P* < 0.001, Fischer’s Exact test). SSRIs, and specifically citalopram and sertraline, were the most frequently prescribed medications for mood stabilisation in the RDD group (19.8% and 12.7% respectively), and significantly more compared to BPAD group (1.60%, *P* < 0.05, Fischer’s Exact test). Sodium valproate was another anticonvulsant and mood stabilising medication that was significantly more prescribed for patients with BPAD in our cohort (20.6% versus 0, *P* < 0.001, Fischer’s Exact test; Supplementary Table 3). In contrast, no group difference was observed for pregabalin, a treatment that is often used in treating anxiety and sleep disorders such as RLS or PLMD and as an adjunctive medication for partial seizures in epilepsy (11.1% versus 6.3%, *P* = 0.393, Fischer’s Exact test).

Perhaps unsurprisingly, patients in our cohort were also frequently treated with medications for sleep disturbances (Supplementary Table 3). In particular, patients with BPAD were more often prescribed with zopiclone relative to depressed patients (14.3% versus 4.8%, *P* = 0.042, Fischer’s Exact test). On the other hand, hypnotic medications such as benzodiazepines including diazepam (4.80% versus 1.60%, *P* = 0.335, Fischer’s Exact test), temazepam (3.20% versus 0.80%, *P* = 0.258, Fischer’s Exact test) and lorazepam (0 versus 1.6%, *P* = 0.553, Fischer’s Exact test) did not differ significantly between patients with BPAD and RDD. Descriptive statistics for other medications are shown in Supplementary Table 3.

## Discussion

We report a strong association between OSA and insomnia, two most prevalent sleep disorders, and BPAD, in a selected cohort of patients from a tertiary sleep clinic. A significant link was also demonstrated between OSA and insomnia and RDD patients, in keeping with previous studies^17–20^. However, this association was significantly less strong for RDD, which could be arguably explained by distinct metabolic profiles of our two cohorts^21^. By way of example, patients with BPAD in our study were reported to be more obese than their RDD counterparts, and they had a higher frequency of comorbid physical conditions including diabetes, hypothyroidism and asthma, which is consistent with previous epidemiological findings^22,23^. Conversely, RDD patients most commonly reported mild sleep problems that did not reach full diagnostic casesness (PSQ). It is of note that investigations were done during patients’ presumed full or partial remission, which arguably also explain lower prevalence of insomnia noted for both cohorts.

High prevalence and incidence of OSA have been previously reported in BPAD^24–26^. In that background, some authors have argued that a reduction in noradrenaline and serotonin delivery to upper airway dilator motor neurons in sleep might contribute to reduced dilator muscle activity and increased upper airway obstruction, resulting in OSA^27,28^. Perhaps consistently, our RDD patients were more often prescribed medications with REM-suppressing properties (e.g. mirtazapine and fluoxetine), which may be theoretically protective against respiratory related arousals and REM-related OSA. Conversely, however, other studies have suggested that chronic management with psychotropic medications can similarly inhibit the serotonergic pathway, and thus increase the upper airway resistance and eventually worsen symptoms in OSA^29,30^. To date, the effects of psychotropic medications on the relationship between REM sleep and OSA in patients with affective disorders remain unclear and thus any inferences remain only conjectural. Moreover, our data did not account for possible non-adherence to the prescribed regimes, which is relatively common amongst the RDD and BPAD patients, and which may further skew any findings^31^.

The primary goal of our study was to investigate differences in sleep disturbances and the sleep macrostructure between BPAD and RDD. We failed to demonstrate any overt differences in their sleep macrostructure (Table 1). Whilst this is still a matter of controversy in the field of neuropsychiatry, it has been previously proposed by some authors that a lack of specific neurophysiological markers in affective disorders might suggest their common underlying neuromechanisms^32,33^. To date, several underlying mechanisms of sleep dysregulation in affective disorders have been proposed. They include disbalance in the cholinergic-aminergic system^34,35^, dysregulated sleep–wake synaptic activity^36,37^ and increased allostatic load^38^; all of which are implicated in the pathophysiology of both BPAD and RDD. Although current findings could not provide robust results for a specific hypothesis building, similarities in sleep architecture parameters may be similarly attributed to the use of antidepressants with REM suppressing or enhancing properties^39,40^, which may reflect the modulatory role of monoamine system in sleep and affective regulation. In this study, patients with BPAD and RDD were commonly treated with SSRIs and SNRIs which help ameliorating depressive symptoms and reducing the elevated amount of REM sleep^41–43^. However, due to the retrospective design of our study, neither causality or directionality of these associations can be deduced.

Another interesting finding of our study is relatively mild severity of recorded OSA in both psychiatric cohorts (Fig. 2), even in the presence of high prevalence of co-morbid obesity. Research evidence to date suggests a close relationship between being overweight and obesity with OSA^44^. However, the extent of this relationship among different population groups, including those with psychiatric co-morbidity, is unclear. Obesity has been in past also linked with higher percentage of hypopneas^45^, and in keeping, this was another striking finding in both of our cohorts (Fig. 3). As already mentioned, due to the retrospective and a real-world nature of our study, any possible mechanistic platform behind these observed phenotypes of OSA in RDD and BPAD remains unclear. For example, theoretically, these findings may reflect, at least in part, modulation of cortical arousal by antidepressant medications taken by our patients. Similarly, milder OSA severity during REM sleep in RDD patients, as observed in our study, could simply reflect an increased use of medications with a REM suppressing effect in this cohort group^46,47^.

In addition, our study highlights several specific clinical issues. For example, the current gold standard treatment of OSA, positive airway pressure (PAP) therapy, is known to be poorly tolerated by majority of patients, and compliance with the treatment is especially challenging in patients with psychiatric co-morbidities. In future, baseline respiratory/sleep biomarkers, such as for instance the HI/AHI ratio in our study, could potentially offer a pivotal clinical insight and allow for a better pathophysiological characterisation of the phenotype of OSA, and accordingly lead to a more personalised treatment^48^. For instance, in our study, we demonstrated higher proportion of the hypopneas in both RDD and BPAD cohorts. Although this remains a matter of some debate^49–51^, similar OSA phenotype has been in past linked to lower PAP pressure requirements for the maintenance of airway’s patency (i.e. to overcome the flow limitation in the dynamic closure of the airway), leading to increased tolerability of treatment^50,52,53^. Comparably, analogous OSA phenotype in a RDD or BPAD patient, may guide an earlier clinical decision towards other types of therapy, such as with mandibular advancement device, known to be better tolerated by some patient groups^45^.

Finally, hypopneas have been historically linked with both adaptive (sleep-promoting) and maladaptive (sleep-instability increasing) cortical arousals^54,55^, with the latter subtype likely associated with a more severe OSA and higher cognitive^56^, metabolic and physiologic deficits^55,57^. Against that background, it is of note that both BPAD and RDD’s affective symptomatology can lead to ‘cognitive’ hyperarousal, which in turn has been shown to increase spontaneous arousals from sleep^58^. Spontaneous arousals of sleep, on the other hand, appear to share the same neurocircuitry and mechanisms to those of respiratory arousals in OSA^59^. Thus, observed OSA phenotype in our patients may reflect a complex interplay of the RDD and BPAD biology, modulated by the idiosyncratic homeostatic and circadian pressure of sleep. Limited evidence to-date also suggests that patients with this particular phenotype of OSA (e.g. with increased overnight HI/AHI ratio and with associated hyper-arousability) may clinically benefit from addition of low doses of sedative medication^60,61^ to their standard treatment protocols (e.g. MAD or PAP). Also, in keeping with promising results shown for OSA with other comorbid sleep disorders^62^, it is tempting to propose that in future, distinct cognitive behaviour therapy courses, especially those that target cognitive hyperarousal levels, and that are further tailored to the specific needs of the BPAD or RDD patients, may achieve similar beneficial results. In future, it will be vital to discern a neural mechanism behind affective symptomatology-driven hyperarousal and its effect on OSA-hypopneas and their physiologic fingerprint. Also, we will need to work out distinct cognitive, affective, and behavioural correlates of this interplay.

In summary, whilst all the findings we report can be arguably attributed to the limitations of our tertiary centre recruitment bias, occupational need, accessibility to health care, higher recorded metabolic^21^ and other comorbidity burden in BPAD cohort, as well as recorded differential treatment regimes, we believe they nonetheless merit further investigation. Importantly, they may provide an important clinical message in regards to a significant health system referral bias. For example, our findings could be taken to suggest that patients with RDD are more likely (more easily) to be referred relatively early in their illness progression, whilst suffering with their relatively mild complaints. Conversely, BPAD patients we investigated were more likely to be on polytherapy and with suspected well established metabolic syndrome, perhaps suggestive of much later referral in the course of their illness, despite their obvious (longer-standing) and significant sleep disorder burden (37.5% of moderate to severe OSA and 34.9% of insomnia for BPAD versus 19.9% and 15% for RDD, respectively). Finally, one should note that our findings are based on only one snapshot in the disease progression timeline of these two complex affective disorders, with no further assessment of the disease severity, and thus no further conclusions in regards to the association with the illness severity or its stage could be drawn. Various stages of BPAD and RDD (e.g. acute, mild, severe, manic episode, depression, remission, comorbid with anxiety and/or other disorders) very likely command differential sleep phenotypes, as of yet not fully understood. Future prospective multicentre studies should consider a multidisciplinary approach, including an active collaboration between psychiatric, internal medicine and sleep physicians, which would allow for more accurate and verifiable assessments of illness progression and help elucidate a viable and meaningful physiologic biomarkers for sleep phenotypes.

## Supplementary Information

Supplementary Tables.
