# Supplementary material for: Profile of sleep disturbances in patients with recurrent depressive disorder or bipolar affective disorder in a tertiary sleep disorders service
Source: Sci Rep. 2023 May 31;13:8785. doi: 10.1038/s41598-023-36083-7 (PMC10232417; doi:10.1038/s41598-023-36083-7)
Supplement: Supplementary file 1 — Supplementary Tables. [file 41598_2023_36083_MOESM1_ESM.docx]

Supplement to: **Profile of Sleep Disturbances in Patients with Recurrent Depressive Disorder or Bipolar Affective Disorder**

## Supplement Outline

1. **Supplementary Results**

Supplementary Table 1. Other (Non-Sleep) Comorbidities

Supplementary Table 2. Pharmacotherapy

Supplementary Table 1. **Other Common Comorbidities.** Group comparisons were conducted using Pearson’s χ^2^ tests for parametric variables and Fischer’s Exact tests for non-parametric variables.

| Comorbid conditions | BPAD | RDD | | χ^2^ | *P* value | |
| --- | --- | --- | --- | --- | --- | --- |
|  | n (%) | | n (%) |  |  |  |
| Obesity | **33(52.4%)** | | **39(31.0%)** | **8.18** | **0.007*** | |
| Diabetes | **9(14.3%)** | | **4(3.20%)** | **-** | **0.011*** | |
| Hypothyroidism | **9(14.3%)** | | **3(2.40%)** | **-** | **0.003*** | |
| Asthma | **7(11.1%)** | | **4(3.20%)** | **-** | **0.044*** | |
| Anxiety | 6(9.50%) | | 9(7.10%) | - | 0.577 | |
| Hypertension | 5(7.90%) | | 10(7.90%) | - | 1.000 | |
| IBS | **5(7.90%)** | | **0** | **-** | **0.004*** | |
| Epilepsy | 4(6.30%) | | 4(3.20%) | - | 0.444 | |
| COPD | 4(6.30%) | | 2(1.60%) | - | 0.097 | |
| Fibromyalgia | **4(6.30%)** | | **1(0.80%)** | - | **0.043*** | |
| Migraine | **4(6.30%)** | | **1(0.80%)** | - | **0.043*** | |
| ADHD | **3(4.80%)** | | **0** | **-** | **0.036*** | |
| Hyperlipidaemia | **3(4.80%)** | | **0** | - | **0.036*** | |
| PD | 2(3.20%) | | 4(3.20%) | - | 1.000 | |
| Chronic pain | 2(3.20%) | | 3(2.40%) | - | 1.000 | |

%, percentage; ADHD, attention deficit hyperactivity disorder; BPAD, bipolar affective disorder; COPD, chronic obstructive pulmonary disease; IBS, irritable bowel syndrome; n, number; PD, personality disorder; RDD, recurrent depressive disorder.

*: *P*<0.05 χ^2^ test.

Supplementary Table 2. Statistically significant correlations of hypopnea index as fraction of AHI in BPAD and RDD, with OSA metrics, sleep macrostructure and demographics. Analysis was performed with Pearson Correlation.

|  | AHI | AHI_NREM_ | NREM2% | WASO | TST | BMI |
| --- | --- | --- | --- | --- | --- | --- |
| HI/AHI  (BPAD) | r=-0.359  P=.044 | r=-0.397  P=.025 | r=0.474  P=.006 | r=-0.177  P=.333 | r=0.072  P=.695 | r=0.043  P=.818 |
| HI/AHI (RDD) | r=-0.413  P=.011 | r=-0.495  P=.002 | r=0.339  P=.040 | r=-0.387  P=.018 | r=0.457  P=.004 | r=1  P=.557 |

BMI, body mass index; BPAD, bipolar affective disorder; HI/AHI, hypopneas index to apnea and hypopneas index; NREM, non-rapid eye movement sleep; RDD, recurrent depressive disorder; TST, total sleep time; WASO, wakefulness after sleep onset.

Supplementary Table 3. **Most Frequently Recorded Pharmacological Treatments.** Group comparisons were conducted using Fischer’s Exact tests.

| Medications | BPAD | RDD | | | χ^2^ | *P* value | |
| --- | --- | --- | --- | --- | --- | --- | --- |
|  | n (%) | | | n (%) |  |  |  |
| Lamotrigine | **17(27.0%)** | | | **1(0.80%)** | **-** | **<0.001*** | |
| Sodium valproate | **13(20.6%)** | | | **0** | **-** | **<0.001*** | |
| Lithium | **12(19.0%)** | | | **3(2.40%)** | **-** | **<0.001*** | |
| Omeprazole | **10(15.9%)** | | | **11(8.70%)** | 2.17 | **0.218** | |
| Mirtazapine | **9(14.3%)** | | | **6(4.80%)** | **5.21** | **0.042*** | |
| Zopiclone | **9(14.3%)** | | | **6(4.80%)** | **5.21** | **0.042*** | |
| Aripiprazole | **8(13.0%)** | | | **0** | **-** | **<0.001*** | |
| Pregabalin | 7(11.1%) | | | 9(7.10%) | 0.85 | 0.409 | |
| Venlafaxine | 6(9.50%) | | | 12(9.50%) | 0.00 | 1 | |
| Levothyroxine | 6(9.50%) | | | 9(7.10%) | 0.33 | 0.777 | |
| Salbutamol | 6(9.50%) | | | 5(4.00%) | - | 0.184 | |
| Quetiapine | 6(9.50%) | | | 4(3.20%) | - | 0.086 | |
| Olanzapine | **6(9.50%)** | | **0** | | **-** | **0.001*** |  |
| Duloxetine | 5(7.90%) | | 4(3.20%) | | - | 0.163 |  |
| Fluoxetine | 3(4.80%) | | | 12(9.50%) | - | 0.393 | |
| Diazepam | 3(4.80%) | | | 2(1.60%) | - | 0.335 | |
| Temazepam | 2(3.20%) | | | 1(0.80%) | - | 0.258 | |
| Citalopram | **1(1.60%)** | | | **25(19.8%)** | **11.80** | **<0.001*** | |
| Sertraline | **1(1.60%)** | | | **16(12.7%)** | **6.33** | **0.013*** | |
| Modafinil | 1(1.60%) | | | 7(5.60%) | - | 0.273 | |
| Lorazepam | 0(0%) | | | 2(1.60%) | - | 0.553 | |

%, percentage; BPAD, bipolar affective disorder; n, number; RDD, recurrent depressive disorder.

*: *P*<0.05 χ^2^ test.
